# Improving local health workers’ knowledge of malaria in the elimination phase—determinants and strategies: a cross-sectional study in rural China

**DOI:** 10.1186/s12936-017-1865-1

**Published:** 2017-05-19

**Authors:** Ruoxi Wang, Shangfeng Tang, Jun Yang, Tian Shao, Piaopiao Shao, Chunyan Liu, Da Feng, Hang Fu, Xiaoyu Chen, Tao Hu, Zhanchun Feng

**Affiliations:** 1 School of Medicine and Health Management, Tongji Medical College, Huazhong University of Science & Technology, Wuhan, Hubei China; 2 Union Hospital, Tongji Medical College, Huazhong University of Science & Technology, Wuhan, Hubei China; 3 Bureau of Disease Prevention and Control, National Health and Family, Beijing, China

**Keywords:** Malaria, Influencing factors, Local health workers, Knowledge level

## Abstract

**Background:**

The current stage of malaria elimination in China requires experienced local health workers with sufficient knowledge of malaria who help to keep the public health system vigilant about a possible resurgence. However, the influencing factors of local health workers’ knowledge level are not fully comprehended. This study aims to explore the factors with heavy impact on local health worker’s knowledge of malaria and propose corresponding suggestions.

**Methods:**

Underpinned by stratified sampling method, a cross-sectional survey was carried out between November 2014 and April 2016. Chi square test was performed to identify the factors with potential influence on health workers’ knowledge level of malaria. Bivariate logistic regression was employed to explore the relationship between the predictors and local health workers’ knowledge level of malaria. Layered Chi square test was used to calculate the homogeneity of the interaction between training approaches and the percentage of participants with high-level knowledge.

**Results:**

The endemic type of county and type of organization played the most significant role in influencing local health workers’ knowledge level regarding malaria in the sample population. The participants from Type 1 and Type 2 counties were 4.3 times (4.336 and 4.328, respectively) more likely to have high-level knowledge of malaria than those who work in Type 3 counties. The probability of having high-level knowledge amongst the participants from county-level facilities (county hospitals and CDCs) were more than 2.2 times higher than those who work in villages. Other socio-demographic factors, such as education and work experience, also affected one’s knowledge regarding malaria. Amongst the six most-used training approaches, electronic material (OR = 2.356, 95% CI 1.112–4.989), thematic series (OR = 1.784, 95% CI 0.907–3.508) and supervision (OR = 2.788, 95% CI 1.018–7.632) were proven with significant positive impact on local health workers’ knowledge of malaria.

**Conclusion:**

Village doctors and who served in Type 3 counties were identified as the ones in urgent need of effective training. Three types of training approaches, including electronic material, thematic series and supervision, were proven to be effective in improving local health workers’ knowledge. Nevertheless, the coverage of these training approaches was still limited. This study suggests expanding the coverage of training, especially the three particular types of training, to local health workers, particularly to the target populations (village doctors and who served in Type 3 counties). Online training, small group discussion and targeted skill development may be the directions for the future development of training programmes.

## Background

Malaria, one kind of human parasitic diseases, has been and still is causing hundreds of thousands deaths globally each year, bringing about serious damage to population health and economy of the areas where it appears [1, 2]. Tracing back to the period between 1960s and 1970s, China used to be one of the countries that deeply suffered from the large-scale outbreaks of malaria [3]. Since the establishment of the National Malaria Control Programme, the Chinese government has made huge efforts into control the spread of the disease [4]. In particular, since the Chinese government successfully built a close collaborative relationship with the Global Fund in 2004, the past 10 years has witnessed an effective control of the malaria endemic [5]. The fight against malaria has moved on to the next phase—the Chinese government initiated the National Malaria Elimination Programme (NMEP) in 2010, with an ambitious goal to completely eliminate malaria by 2020 [6].

Nevertheless, the fight against malaria is a global task that no one country can do this alone. The increase in China’s cross-border trade with South Asian countries, including Laos and Myanmar which are malaria endemic countries, has inevitably increased the threat of imported cases [5]. Similarly, the economic globalization has led to a dramatic increase in the number of Chinese migrant workers in sub-Saharan Africa, which also contributed to a rapid increase of imported cases [2, 7]. It has become a consensus that the imported cases and the left hard-to-reach indigenous cases are the major challenges in the current elimination phase, and therefore, this calls for new strategies for new challenges [8, 9].

Since 2010, malaria cases have become increasingly rare (3078 cases vs. 1.4 billion population in 2014 [4]), which indicates that previous proactive surveillance strategies, such as mass malaria drug administration, although used to be effective, may not be the best choice when cost-effectiveness is considered. Instead, passive surveillance has become a favoured choice for identification and containment of malaria transmission [9–11]. Under such circumstances, local health workers, who involved in the diagnosis and treatment of malaria, have becoming increasingly important in terms of promptly identifying malaria patients and providing targeted treatment before they lead to transmission [12]. Various studies have highlighted the importance of having skilful health workers at all levels [13], and providing sufficient training accordingly, especially to health workers at the community/village level, where the high-risk groups tend to live [5, 14]. However, what factors influence local health workers’ knowledge of malaria and whether the current training approaches have served their purposes have not been fully discussed. Therefore, this study was conducted to explore the influencing factors of local health workers’ knowledge of malaria and the current effect of training approaches in five malaria endemic regions, which may provide the local governments with implications for their human resource development strategies.

## Methods

### Study population and sampling

This study was based on data derived from a cross-sectional survey carried out in five malaria endemic regions, including Guangxi autonomous region, Hainan Province, Hubei Province, Henan Province and Anhui Province between November 2014 and April 2016. Counties in sampled provinces were divided into three types (Type-1, Type-2, Type-3) according to the incidence rate of malaria (set in the NMEP). Counties were randomly selected according to their malaria-endemic type in every sampled province; three townships were randomly selected from each sampled county; and three villages were randomly selected from each sampled township. Local health workers, including frontline public health staff directly engaged in malaria prevention and control of Centers for Disease Control and Prevention (CDCs) at the county level, clinicians, microscopists (at county and township hospitals) and village doctors, were involved in this study.

With the local malaria cases eliminated in the county areas, the number of frontline staffs has been shrinking. It is common for one CDC to hire 3–5 staff to carry out activities directly related to malaria control. County hospitals normally employ approximately 8 staff (including clinicians, microscopists and public health practitioners) to diagnose and treat patients with malaria. Cluster sampling method was conducted in CDCs and hospitals at the county level. Township hospitals also carry out malaria tests within their jurisdiction. Therefore, three health workers were randomly selected from the three groups (one from each group) at each township hospital. All village doctors from the sampled villages were selected. Finally, a total of 407 responses were considered valid.

### Variables and measures

The surveys were carried out by trained medical postgraduates at the School of Medicine and Health Management of Tongji Medical College, Huazhong University of Science and Technology. The structured questionnaire used for survey covers information from three main aspects: relevant socio-demographic information, knowledge level of malaria and types of training programme received. In the questionnaire, 17 close-ended questions (include single and multiple choices) were set to evaluate the knowledge level regarding malaria, covering foundation knowledge, symptom, aetiology and treatment and control/prevention. For single-choice questions, each correct answer was scored as 1; for multiple-choice questions, each correct answer was scored as 1/n (n = total number of correct answers); score 0 was assigned to incorrect answers and “do not know” answers. The accurate rate of these 17 questions was used to measure the knowledge level. The level of knowledge was dichotomized as “high-level” (accuracy ≥60%) and “low-level” (accuracy <60%) [15].

Previous studies reveal that the quality of health workers are associated with socio-demographic factors, including education, level/type of organization, years of work experience, training opportunities [16, 17]. This indicates the potential relationship between local health workers’ knowledge of malaria and these socio-demographic factors. This study therefore took into account seven key characteristics, including sex, age, education, type of organization, endemic type of county, work experience and qualification.

During the preliminary investigation, six most-used types of training were found: (1) supervising authorities (provincial/municipal health and family planning committees) organize lectures. Nationally-renowned speakers are often invited to deliver presentations on a broad topic relating to malaria management while audiences often come from different posts, including public health practitioners, microscopists and clinicians; (2) local healthcare facilities organize training sessions regarding malaria according to their own practical needs; (3) health workers receive electronic material from different sources (e.g. local healthcare facilities, supervising authorities, CDCs, etc.); (4) provincial and county CDCs organize training sessions regarding practical skills, especially skills related to making diagnosis with microscope; (5) local health workers apply for further education programmes through which they are sent to work in higher-level hospitals for a certain period of time. During this period of time, they have the opportunities to practice under the supervision of experienced professionals; (6) supervising authorities inspect local healthcare facilities in terms of their daily practice and make corresponding comments to help local health facilities make improvement. Therefore, the types of training were categorized as lecture, thematic series, electronic material, skill development, further education/visiting and supervision.

### Statistical analysis

Epidata 3.1 (USA) was employed to enter and document data gathered through the questionnaires. Data were double entered. Input errors were identified and corrected during the data screening process. Statistical Package for Social Science (SPSS) v22.0 was employed to perform statistical analysis of the gathered data. Cross-tabulation was used to summarize the characteristics of categorical and dichotomy variables. Chi square test was performed to test the differences in the distribution of “high-level” and “low-level” responses across the subgroups of each characteristic. Bivariate logistic regress was adopted to explore the correlation between the potential indicators and the knowledge level of the participants. P value <0.05 was considered statistically significant. Potential explanatory predictors with statistically significance were enrolled in the regression model. Wald statistic with backward stepwise selection method was performed to identify the differences between the subgroups in terms of their impact on local health workers’ knowledge level of malaria. The homogeneity of the interaction between training methods and the percentage of participants with high-level knowledge were calculated by layered Chi square test.

## Results

### Sample characteristics

Regarding the basic characteristics of the respondents as shown in Table 1, there was no significant difference in gender, level of education or affiliation. In addition, most of the respondents were between the ages 31 and 50 (67.3%). A total of 63.6% participants served as local health workers for more than 10 years. Participants who had no qualification, junior qualification and middle qualification, respectively, accounted for approximately 30% of the total sample population, leaving 10.3% with senior qualification. Participants who came from Type 2 and Type 3 counties were slightly more than those from Type 1 counties. In terms of training, 80% of the respondents claimed they had attended at least one training session in the past year. According to the training approaches, the most commonly used one was lecture, with 61.4% participants who claimed they had attended at least one lecture. Participants who had received electronic material, thematic series or skill development training respectively accounted for 51.1%, 54.2% and 43.6% of the sample population. In sharp contrast to the above training approaches, only 10.6% of them had an experience of further education/visiting and 19.0% had been trained in a form of supervision.

**Table 1:** Factors with potential impact on health workers’ knowledge of malaria

Characteristics	n	%	Low-level of knowledge (n = 114)	High-level of knowledge (n = 293)	*χ* ^2^	p
Sex					0.017	0.896
Male	233	57.5	57.0	57.7		
Female	172	42.5	43.0	42.3		
Age					0.562	0.905
≤30	58	15.8	15.1	16.1		
31–40	126	34.3	33.0	34.9		
41–50	121	33.0	35.9	31.8		
>50	62	16.9	16.0	17.2		
Education					4.69	0.096
High school and lower	108	30.9	40.0	27.8		
College	119	34.1	28.9	35.9		
Bachelor and above	122	35.0	31.1	36.3		
Type of organization					15.386	0.002
County hospitals	123	30.7	27.7	31.9		
CDC	105	26.2	19.6	28.8		
Township hospital	81	20.3	17.0	21.6		
Village clinic	91	22.8	35.7	17.7		
Work experience					1.024	0.796
≤5	62	15.3	15.8	15.0		
6–10	86	21.1	20.2	21.5		
11–20	112	27.5	30.7	26.3		
>20	147	36.1	33.3	37.2		
Qualification					8.41	0.038
No qualification	121	29.8	37.7	26.7		
Junior	123	30.3	22.8	33.2		
Middle	120	29.6	32.5	28.4		
Senior	42	10.3	7.0	11.7		
Endemic type of county					43.87	<0.001
Type 1	109	26.8	18.4	30.0		
Type 2	166	40.8	24.6	47.1		
Type 3	132	32.4	57.0	22.9		
Training					3.96	0.047
No	81	20.0	26.3	17.5		
Yes	324	80.0	73.7	82.5		
Training 1-lecture					1.02	0.312
No	139	38.6	42.9	37.0		
Yes	221	61.4	57.1	63.0		
Training 2-electronic material					0.94	0.333
No	176	48.9	53.1	47.3		
Yes	184	51.1	46.9	52.7		
Training3-thematic series					6.937	0.008
No	165	45.8	57.1	41.6		
Yes	195	54.2	42.9	58.4		
Training 4-skill development					0.004	0.950
No	203	56.4	56.1	56.5		
Yes	157	43.6	43.9	43.5		
Training 5-further education/visiting					1.59	0.207
No	321	89.4	92.8	88.2		
Yes	38	10.6	7.2	11.8		
Training 6-supervision					2.70	0.100
No	290	81.0	86.6	78.9		
Yes	68	19.0	13.4	21.1		

### Differences in the level of knowledge regarding malaria

Table 1 compares participants with good and poor medical knowledge regarding malaria according to their socio-demographic characteristics and type of training they had received. Generally speaking, amongst 407 participants, 71.9% of them (n = 293) had obtained “high-level knowledge”. There was no significant difference in the degree of medical knowledge regarding malaria between different gender or age groups. Participants with a college degree or above showed higher level of medical knowledge of malaria than those who had a high school degree or under. Similarly, participants with qualifications were more likely to have better knowledge than those without a qualification. Participants from hospitals at township level or above, CDCs and county hospitals did much better in the test than village doctors. Compared with those who had worked for no more than 5 years and more than 10 years, participants with 6–10 years’ experience were more likely to have higher level of knowledge regarding malaria. A comparatively high percentage of participants with “high-level knowledge” was detected in both Type 1 and Type 2 county groups whereas the percentage was significantly lower in the Type 3 county group.

Regarding the general impact of training, for participants who had received at least one type of training approaches with the past year, a higher percentage of them had good knowledge of malaria, whereas participants who had not received any kind of training were more likely to have low degree of medical knowledge regarding malaria. Similarly, participants who had received any single training approach, including lecture, electronic material, thematic sessions and further education/visiting or supervision, did better in the test than those who had not done so.

### Predictors affecting knowledge level

#### (1) *Endemic type of county and type of organization*

**Fig. 1:**
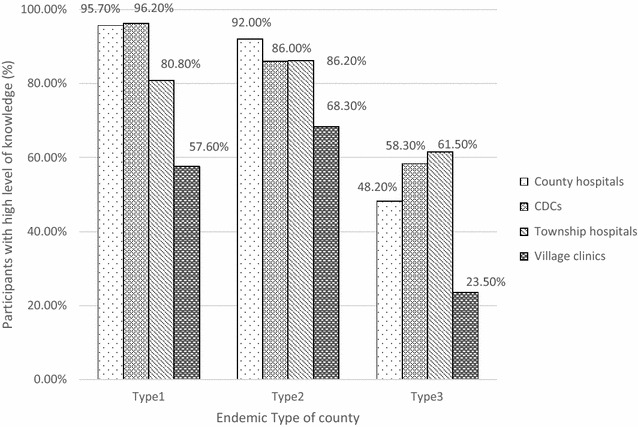
Distribution of participants with high-level of knowledge regarding malaria across endemic types of counties

A binary logistic regress was adopted to examine the potential predictors affecting health workers’ knowledge level of malaria. Factors with significant impact on the degree of knowledge regarding malaria were reported in Table 2. Compared with those from Type 3 counties, health workers from Type 1 and Type 2 counties were 4.3 times more likely (4.336 times and 4.328 times, respectively) to obtain high-level knowledge regarding malaria. Health workers from county hospitals and CDCs had significantly higher level of knowledge than those from village clinics (OR = 11.51, 95% CI 3.709–35.765; OR = 9.008, 95% CI 3.142–25.289), whereas the percentage of participants with high-level knowledge amongst those who worked at township hospitals was also higher than those from village clinics, though the difference was not significant. Figure 1 outlines the percentage of participants with “high-level knowledge” regarding malaria according to the endemic type of county and the type of organization. The outcome complies with the corresponding outcome listed in Table 2, confirming that for participants from any type of organization, the percentage of participants with high-level knowledge regarding malaria decreased from Type 1 to Type 3 counties, although the degree of differed slightly from one to another. Participants from village clinics had the lowest level of knowledge when compared with those from the other three types of organizations.

**Table 2:** Predictors of local health worker’s knowledge level of malaria

Predictors	Reference	B	p	OR	95% CI	
					Lower	Upper
Endemic type of county	Type-3					
Type 1		4.336	<0.001	76.402	21.539	271.009
Type 2		4.328	<0.001	75.760	23.259	246.765
Type of organization	Village clinics					
County hospitals		2.444	<0.001	11.517	3.709	35.765
CDCs		2.198	<0.001	9.008	3.142	25.289
Township hospitals		0.244	0.654	1.275	0.440	3.696
Education	High school and below					
College		1.142	0.009	3.13	1.331	7.377
Undergraduate and above		1.114	0.015	3.047	1.240	7.489
Work experience	≤5 years					
6–10 years		1.078	0.089	2.939	0.849	10.164
11–20 years		−0.199	0.709	0.819	0.287	2.337
>20 years		−0.619	0.254	0.539	0.186	1.560
Training						
Electronic material	No	0.857	0.025	2.356	1.112	4.989
Thematic series	No	0.579	0.093	1.784	0.907	3.508
Supervision	No	1.025	0.046	2.788	1.018	7.632

### (2) *Education and work experience*

The probability of obtaining high-level knowledge amongst who had a college or undergraduate degree was significantly higher than that amongst those who had no more than a high school degree (OR = 3.13, 95% CI 1.331–7.377; OR = 3.047, CI 1.240–7.489). When compared by work experience, health workers with 6–10 years’ experience seemed to be the group with the highest proportion of high-level of knowledge regarding malaria. The group with the second highest level of knowledge belonged to those who had no more than 5 years’ working experience. The percentages of participants with high-level knowledge amongst those with 11–20 years’ and more than 20 years’ work experience were lower than those from the above two groups (B = −0.199, OR = 0.819, 95% CI 0.287–2.337; B = −0.619, OR = 0.539, 95% CI 0.186–1.560).

### (3) *Training approaches*

The results also indicated that three out of six training approaches had significantly positive impact on participants’ knowledge level regarding malaria, including electronic material (OR = 2.356, 95% CI 1.112–4.989), thematic series (OR = 1.784, 95% CI 0.907–3.508) and supervision (OR = 2.788, 95% CI 1.018–7.632). As shown in Tables 3, 4 and 5, layered Chi square test was employed to compare the percentage of participants with high-level knowledge between the trained participants and those had not received training sessions. In almost all relevant subgroups (factors listed in Table 2), the participants who had received electronic materials were more likely to have high-level of knowledge regarding malaria than those who had not. It was the same case between participants who had attended thematic series and those who had not, as well as between those who had been supervised and those who had not been, although the degree of difference varied.

**Table 3:** Malaria knowledge level between participants within and beyond the coverage of electronic material

Predictors	No electronic material received		Electronic material received		*χ* ^2^	p
	No. with high-level knowledge regarding malaria/total no.	%	No. with high-level knowledge regarding malaria/total no.	%		
Endemic type of county					36.379	0.00
Type 1	38/47	0.81	40/47	0.85		
Type 2	60/73	0.82	60/72	0.83		
Type 3	26/56	0.46	38/65	0.58		
Type of organization					14.467	0.00
County hospitals	48/65	0.74	30/42	0.71		
CDCs	45/56	0.80	34/40	0.85		
Township hospitals	16/24	0.67	40/49	0.82		
Village clinics	12/26	0.46	32/51	0.63		
Education					3.452	0.17
Undergraduate and above	46/59	0.78	37/50	0.74		
College	42/57	0.74	41/48	0.85		
High school and below	28/44	0.64	35/49	0.71		
Work experience					0.677	0.97
≤5 years	18/27	0.67	19/25	0.76		
6–10 years	22/31	0.71	32/41	0.78		
11–20 years	34/47	0.72	37/54	0.69		
>20 years	50/71	0.70	50/64	0.78

**Table 4:** Malaria knowledge level between participants within and beyond the coverage of thematic series

Factors	No thematic series attended		Thematic series attended		*χ* ^2^	p
	No. with high-level knowledge regarding malaria/total no.	%	No. with high-level knowledge regarding malaria/total no.	%		
Endemic type of county					36.379	0.00
Type 1	46/54	0.85	32/40	0.80		
Type 2	41/55	0.75	70/90	0.88		
Type 3	22/56	0.39	42/65	0.65		
Type of organization					14.467	0.00
County hospitals	35/53	0.66	43/54	0.80		
CDCs	39/50	0.78	40/46	0.87		
Township hospitals	18/28	0.64	38/45	0.84		
Village clinics	16/32	0.50	28/45	0.62		
Education					3.542	0.17
Undergraduate and above	34/47	0.72	49/62	0.79		
College	32/48	0.67	51/57	0.90		
High school and below	31/47	0.66	32/46	0.70		
Work experience					0.677	0.88
≤5 years	22/30	0.73	15/22	0.68		
6–10 years	20/31	0.65	34/41	0.83		
11–20 years	33/51	0.65	38/50	0.76		
>20 years	34/53	0.64	66/82	0.81

**Table 5:** Malaria knowledge level between participants within and beyond the coverage of supervision

Factors	No supervision received		Supervision received		*χ* ^2^	p
	No. with high-level knowledge regarding malaria/total no.	%	No. with high-level knowledge regarding malaria/total no.	%		
Endemic type of county					37.082	0.00
Type 1	58/72	0.81	19/20	0.95		
Type 2	108/131	0.82	12/14	0.86		
Type 3	40/87	0.46	24/34	0.71		
Type of organization					13.284	0.00
County hospitals	64/90	0.71	14/17	0.82		
CDCs	60/74	0.81	18/21	0.86		
Township hospitals	38/53	0.72	18/20	0.90		
Village clinics	40/68	0.59	4/8	0.50		
Education					3.252	0.20
Undergraduate and above	73/96	0.76	10/13	0.77		
College	64/84	0.76	19/21	0.91		
High school and below	54/79	0.68	8/12	0.67		
Work experience					0.576	0.90
≤5 years	28/40	0.70	9/11	0.82		
6–10 years	37/51	0.73	17/21	0.81		
11–20 years	60/87	0.69	11/14	0.79		
>20 years	81/112	0.72	18/22	0.82		

## Discussion

Given the current lack of comprehensive research regarding the knowledge regarding malaria of the local health workers who are involved in identifying and treating patients with malaria, this study was carried out to identify the factors influencing their knowledge of malaria in this elimination stage across malaria-endemic counties in China. The study results illustrate that the knowledge level of malaria is closely related to the background of participants, including the endemic type of county, type or organization, education and work experience, whereas it is also influenced by the use of appropriate training approaches, including electronic material, thematic series and supervision.

### Various effective strategies had helped improve health workers’ knowledge of malaria

This study shows that the vast majority of the participants had high level of knowledge regarding malaria in a context of low incidence. This may have benefited from the great attention the Chinese government has paid in recent years. Policies such as requiring all health facilities in malaria endemic counties (MECs), including county hospitals and township hospitals, to take malaria (blood) tests for all patients with unexplained fever have consolidated local health workers’ knowledge through differential diagnosis of malaria. In particular, the comparison on working experience reveals that participants with less than 10 years’ work experience were more likely to have solid knowledge of malaria than those who had worked more than 10 years. This difference may be brought about by the collaboration between the Chinese government and the Global Fund. Between 2004 and 2014, this 10-year partnership has helped build a public health system with high-level of transparency and greater integration of sophistically developed strategies [18]. The nationwide application of advanced interventions, such as the “1-3-7” model [19, 20], have provided frontline health workers with knowledge about how to effectively treat patients and control transmission in compliance with international standards. The richer the experience with these advanced intervention activities, the better the awareness and knowledge. The interviews were carried out with local health workers, the changes brought about by the Global Fund have been repeated mentioned and regarded as one factor that helped improve their knowledge of malaria.

### Health workers in Type 3 counties and village doctors require greater attention

The endemic type of county was proved to be the factor that influenced the knowledge level of the participants the most. Participants in Type 1 and 2 counties had significantly higher degree of knowledge regarding malaria than those in Type 3 counties. Indeed, experience with malaria may be one reason—participants in Type 1 and 2 counties may have encountered patients with malaria and the corresponding treatment and control interventions helped familiarize themselves with this kind of disease. For most Type 3 counties, there had been no malaria cases for several consecutive years and, therefore, the lack of experience with patients with malaria may have resulted in their unfamiliarity with professional knowledge, especially that related to symptom and aetiology. It may have also been influenced by government mind-set. During investigation, it found that compared to Type 1 and Type 2 counties, local authorities in Type 3 counties had more tight budget regarding malaria control related activities, such as health promotion activities and training programmes, since malaria control was no longer a prioritized task in these areas. In some sampled areas, malaria was no longer a top priority in training programmes as it had been. Rather, it became one of the topics mentioned in the lectures on infectious diseases. Indeed, it is quite often for local authorities to adjust their priority lists when malaria cases become increasingly rare, which results in less funding and other resources granted. However, this has been proved of great risk, since the over 90% of the resurgences in the past 80 years were attributed, at least in part, to the weakening of malaria control efforts, particularly resource constraints [21].

Type of organization and education experience were considered as two other factors with significant impact on local health workers’ knowledge. In light of the context of a severe maldistribution of health professionals in China, health workers who serve as village doctors tend to be the least educated ones equipped with the poorest resources [16]. The result shows significant lower percentage of participants with high-level of knowledge in this group than in other three groups. In particular, amongst village doctors who serve in Type 3 counties, only 23.5% of them have high level of knowledge regarding malaria, which is worrying. This confirms prior studies which revealed frontline health workers’ lack of awareness and knowledge, which has led to their misdiagnosis of malaria with upper respiratory tract infections or gastritis diseases [22]. However, in the current stage, the high-risk groups (Chinese migrant labours in Africa and the hard-to-reach population) tend to live in rural and remote areas [10], and this study discovered that it is not uncommon for them to delay or do not seek treatment due to their underestimate of the risks and concern of cost. These groups of patients are at higher risk of developing severe forms and are more likely to infect other people [23]. The current situation requires village doctors, the “gatekeepers”, to have higher awareness and be more skilful to identify suspicious cases and make prompt referral in order to get patients managed before transmission. This reminds the importance and urgency to provide health workers, especially who serve at the ground level and who work in Type 3 counties, with sufficient training to improve their awareness and capacity on diagnosis [5, 22].

### Appropriate training approaches have positive impact on health workers’ knowledge of malaria

In terms of training approaches, the results of logistic regression indicate three types of training, including electronic material, thematic series and supervision, with significant impact on local health workers’ knowledge of malaria. This conforms to the interview findings, which indicated that compared to traditional training approaches, such as lecture and thematic series which require long time travel and a particular time slot, local health workers prefer to use internet reading electronic material in their spare time. Electronic materials enable them to review knowledge anytime anywhere, which is more helpful in consideration of their tight working schedule. This indicates the potential of developing remote training systems/approaches which provide a friendlier learning environment (and also more opportunities) for local health workers, particularly who work in remote areas.

Interestingly, the results indicate that thematic series had significant impact on the degree of knowledge whereas lecture did not. The difference in content may be the reason: compared to lectures, thematic series are organized by local health facilities (including county and township hospitals, county CDCs), attended by their own staff and with topics focusing on practical and targeted knowledge. In addition, with a smaller amount of audiences, thematic series provide a more informal and friendly environment where local health workers have more opportunities to ask questions and exchange views with each other. These two factors may have contributed to the effectiveness of training. This suggests that in this stage, it may still be necessary to keep malaria as a key theme and to help local health workers solve specific problems they encounter in their daily practice.

Supervision is the last training approach considered effective in improving local health workers’ knowledge of malaria. Supervision is more than a traditional inspection in which superior authorities inspect healthcare facilities in terms of the quality of service provision since investigators, who are professionals, often provide technical support while supervising or comments for improvement. On the one hand, the inspection/evaluation itself helps local healthcare facilities/health workers raise attention to malaria control and management. On the other hand, the technical support and comments provide local health workers with guidelines on how to improve their skills. The onsite investigation findings also suggested that targeted comments and technical support are helpful and welcome by local health workers. It is worth noting that supervision was much less used than inspection in the sample sites since almost all participants had experience with inspection in the past year whereas only around 19% of the participants had been supervised in the same period of time. Under such circumstances, supervision, as an effective approach, may be worth conducting in a broader range.

### Limitations

This cross-sectional study only considered villages in five regions, whereas Yunnan, one province considered as classical malaria endemic regions, was not sampled. This may limit the generalizability of the research findings. In addition, the dependent variables were measured through close-ended questions. This may allow participants to guess correct responses through the process of elimination.

## Conclusions

Experienced health workers who are able to promptly diagnose malaria cases and control transmission are considered as the mainstay of the passive surveillance system and even the NMEP. This study was carried out with an aim to explore the correlation between local health workers’ knowledge level of malaria and their socio-demographic characteristics as well as the currently most-used training approaches. The endemic type of county and type of organization played most significant role in influencing local health workers’ knowledge regarding malaria in the sample population. The results revealed that local health workers who serve in Type 3 counties and who are village doctors are in urgent need of effective training in consideration of their knowledge level regarding malaria as well as their responsibilities. Amongst the most-used training approaches, electronic material, thematic series and supervision were proven to be effective in improving local health workers’ knowledge. However, the coverage of these three training approaches is still limited: only slight more than 50% participants had received electronic materials or attended thematic series and less than 20% of the participants have been supervised in the past year. This reminds policymakers of the importance of keeping sufficient attention to the malaria elimination programme, in particularly, ensuring sufficient financial input into the training programme. This study also suggests expanding the coverage of training, especially the three particular types of training, to local health workers, particularly to the target populations (village doctors and who serve in Type 3 counties). Online training, small group discussion and targeted skill development may be the directions for the future development of training programmes.

## Abbreviations

NMEP: National Malaria Elimination Programme
CDC: Centers for Disease Control and Prevention
CI: confidence interval
